# Analysis of Gender Perceptions in Health Technology: A Call to Action

**DOI:** 10.1007/s10439-020-02478-0

**Published:** 2020-02-20

**Authors:** Lyn Denend, Stacey McCutcheon, Mike Regan, Maria Sainz, Paul Yock, Dan Azagury

**Affiliations:** Stanford Byers Center for Biodesign, Stanford University, 318 Campus Drive, E100, Stanford, CA 94305 USA

**Keywords:** Diversity, Women, Gender equality, Job satisfaction, STEM, Innovation, Biomedical engineering, Medical technology, Medtech

## Abstract

Gender diversity has been linked to positive business results. Yet limited data exist to characterize the gender landscape in health technology, a field that draws employees from both biomedical engineering and medicine. To better understand the state of gender diversity in this industry, we developed a survey to explore leadership representation and perceptions of workplace equality, job satisfaction, and work-life balance. Data from 400 + health technology professionals revealed that women are significantly underrepresented in senior leadership and that men and women experience the workplace differently. Men believe in greater numbers than females that senior leaders are focused on recruiting and promoting women, promotion criteria are equitable, and the major barrier to leadership roles for women is work/family balance. In contrast, women perceive a less meritocratic and inclusive workplace in which their ability to rise is hampered by exclusion from influential communication networks and stereotyping/bias. Perhaps as a result, more than one-third of female respondents are considering leaving their current jobs, citing dissatisfaction with management and a desire for greater advancement opportunities. This study highlights significant gender perception differences in health technology that require further study and proactive remediation for the field to fully realize the benefits of gender diversity.

## Introduction

Numerous studies have determined that gender diversity is a business imperative.^21^ It is linked to positive financial performance,^17^ ensures that the most talented people contribute at the highest levels, and conveys important benefits, including catalyzing and sustaining innovation^20^ and increasing the “collective intelligence” of the organization.^28^

The US healthcare industry seemingly outperforms the rest of corporate America on gender diversity. Depending how healthcare is defined, women make up 50%^3^ to 75%^19^ of the workforce, and the sector has better female representation at all levels of leadership than other US industries.^3^

The high-technology industry, by comparison, continues to struggle with a significant gender gap—its workforce is only about one-quarter female.^26^ While this sector is taking steps to address its unfavorable track record on gender diversity,^24^ the number of women in high-tech has not risen appreciably despite the fact that employment opportunities are abundant and jobs are generally high paying.^7^

Health technology, a growing field that includes medical device, device-based diagnostic, digital health, and health information technology companies, sits squarely between the healthcare and high-technology industries. However, its gender landscape remains obscure as it has not been extensively examined. In most research studies, health technology is grouped with larger healthcare sectors (payers and providers), other life sciences fields (pharmaceuticals, biotechnology), or even included in high-technology rather than investigated separately.^3^,^7^

Efforts have been made to evaluate gender diversity in the disciplines that feed health technology employment, most notably biomedical engineering and medicine. Prior studies have shown that the pipeline of women in these two specialties is robust. University-based biomedical engineering programs have better gender parity than almost any other engineering field. In 2017, women earned 44% of bachelor’s degrees, 43% of master’s degrees, and 39% of doctoral degrees in this discipline.^29^ Medicine is also highly attractive to women, who make up 36% of practicing physicians.^22^ And, for the first time in 2019, women comprise the majority of enrolled medical students.^1^

However, these “pipeline” figures do not tell the whole story. Even though the number of women entering biomedical engineering and, more broadly, science, technology, engineering, and math (STEM) fields is growing, men continue to outnumber women, especially in the leadership ranks.^15^ Multiple studies of females working in STEM jobs have posited that inhospitable culture and bias (rather than pipeline issues or personal choices) cause women to vacate STEM positions.^10^,^15^,^25^ Similarly, in medicine, women remain scarce in the procedural fields like surgery that are a natural fit for those with an interest in health technology innovation. In 2017, women represented less than 25% of practitioners across 10 surgical specialties.^14^ The same year, only 9% of US fellows in interventional cardiology, a field defined by technology innovation, were women.^30^

Against this unclear and even contradictory backdrop, we initiated a survey to characterize the gender landscape specifically within health technology. Our goal was to better understand the industry’s current performance on factors such as female representation in leadership and perceptions of gender dynamics, workplace equality, job satisfaction, and work-life balance.

## Materials and Methods

The survey was designed to collect perceptual data of respondents’ experiences across companies in the health technology workplace to strengthen our understanding of gender dynamics in the field and improve the treatment of this subject in our educational programs. It included 104 questions in seven sections—demographics, industry experience, career path, mentoring, workplace perceptions, family life, and fundraising. Given our focus on gender-related issues, we did not collect data on ethnicity or race.

We were particularly interested in understanding gender dynamics in small and mid-sized companies for three reasons. First, these organizations are generally considered the primary drivers of innovation in our field. Second, a significant portion of our trainees launch or take positions in small and mid-sized companies. And, third, these organizations often lack substantial resources to launch the types of diversity and inclusion initiatives that are emerging within larger corporations in health technology.

To access individuals employed by small and mid-sized health technology companies, we asked the two largest trade organizations, the Medical Device Manufacturers Association (MDMA), which explicitly focuses on companies of this size, and AdvaMed Accel, the subgroup of the Advanced Medical Technology Association that serves medical technology companies with revenues under $100 million, to email the survey to their members. We also asked two other groups to send it: MedtechWomen, a California-based organization whose members we felt would be particularly interested in the survey topic, and the Stanford Biodesign Alumni Association, whose constituents have completed our fellowship. In total, these lists included 6548 individuals with some unknown (but potentially significant) overlap among them.

Using the Qualtrics platform (Qualtrics, Provo, UT), the survey was shared with individuals (as described) between December 17, 2018 and March 4, 2019. Each organization was asked to send an initial email and a second reminder. Respondents were offered a copy of the survey results (post publication) as an incentive for completion. No other inducements were provided.

Survey data from Qualtrics were exported into Microsoft Excel and reformatted for upload into Stata/IC 11.2 for Windows. All data were anonymized prior to being provided to the authors, and data analysis was performed under a waiver from Stanford’s Institutional Review Board. Comparisons between proportions were tested by Pearson’s Chi squared, with statistical significance at *p* < 0.05.

## Results

Survey responses were received from 403 unique individuals. Respondents were 37.7% male (*n* = 152) and 62.3% female (*n* = 251). They varied in age from under 30 to over 60, with a majority (57%, *n* = 230) in the 40–59 age range. 80.1% were married (*n* = 323), 12.4% had never been married (*n* = 50), and 7.4% (*n* = 30) were separated or divorced. 73.2% (*n* = 295) had children.

When asked to rate factors that influenced their choice of a career in medtech, most respondents (79.4%, *n* = 320) said that the desire to help people and improve healthcare was a major motivator. The next most frequently cited motivator was the desire for challenging work (57.3%, *n* = 231).

**Figure 1:**
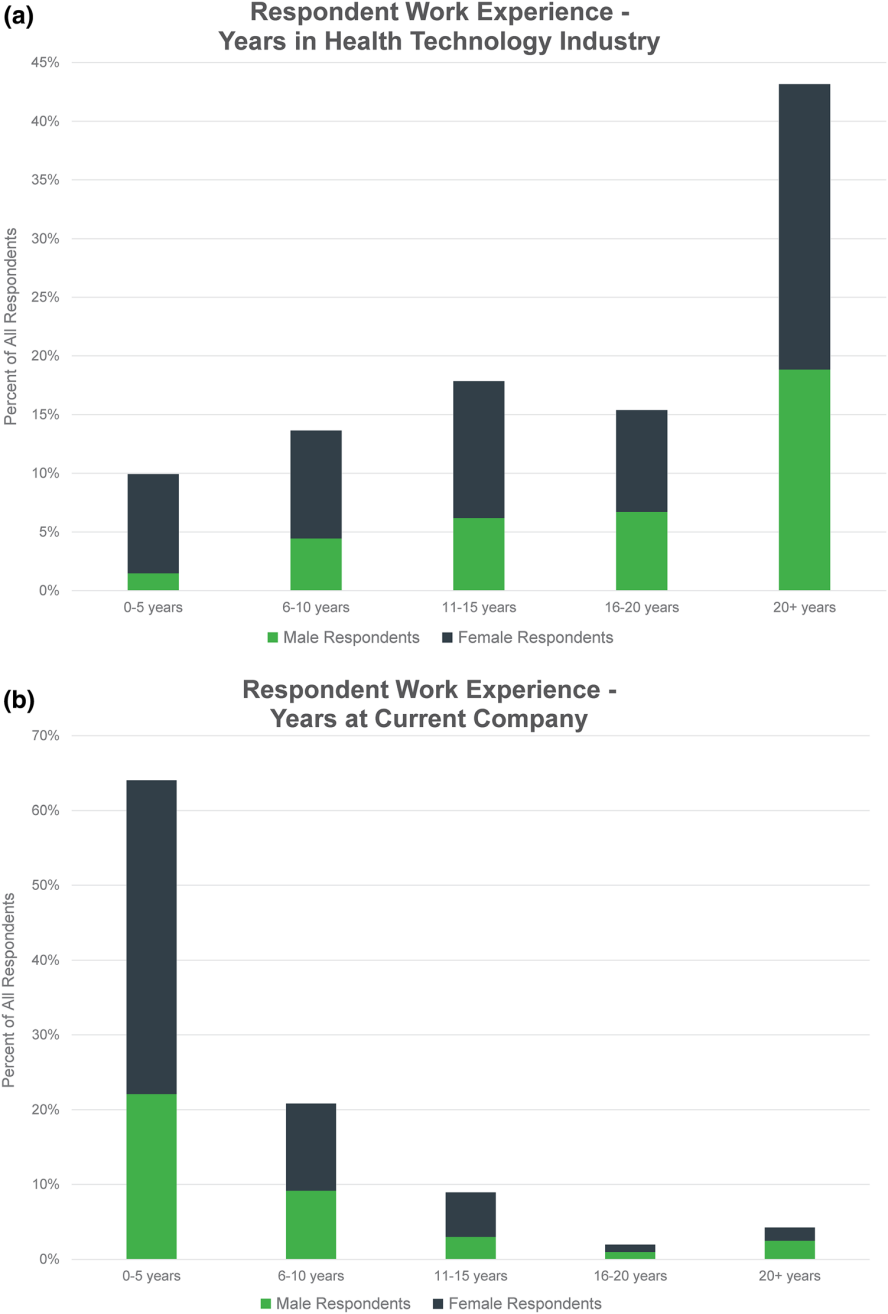

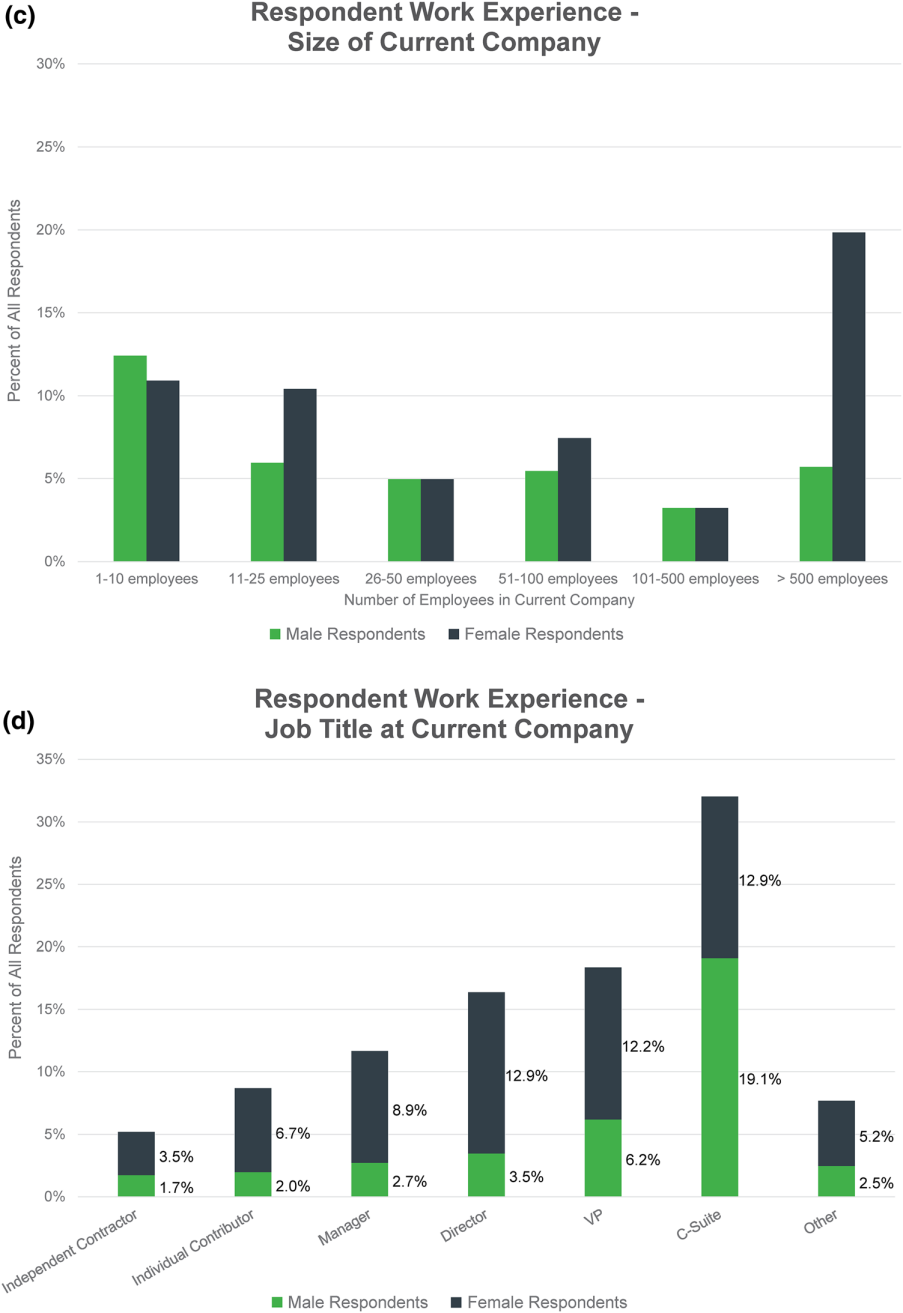
(a) Respondents were asked, “How many years have you worked in the health technology industry?” (b) Respondents were asked, “How long have you been with your current company?” (c) Respondents were asked, “How big (number of employees) is your current company?” (d) Respondents were asked, “What is your current position/role?” and given the listed options from which to choose.

Respondents had worked in health technology for an average of 17.2 years and had been with their current company for an average of 5.8 years (Figs. 1a and 1b).

The companies where respondents were currently employed ranged from very small to very large as follows (Fig. 1c): 23.3% 1–10 employees (*n* = 94), 16.4% 11–25 employees (n = 66), 9.9% 26–50 employees (*n* = 40), 12.9% 52–100 employees (*n* = 52), 11.9% 101–500 (*n* = 48), and 25.6% more than 500 (*n* = 103).

The proportion of male and female respondents varied by current company size (*p* < 0.001). Men were disproportionately represented in the smallest companies, with 33.0% (*n* = 50) working at those with 10 employees or less. Women were more likely to work in large companies, with 32.0% (*n* = 80) holding positions at organizations with more than 500 employees.

5.2% of respondents were currently employed as independent contractors, 8.7% were individual contributors (i.e., no supervisory responsibilities), 11.6% were managers, 16.4% were directors, 18.4% were VPs, 32.0% held C-suite titles (executive-level positions such as CEO, CFO, COO), and 7.7% selected “other” (Fig. 1d). A disproportionate number of all men completing the survey were in C-suite positions (50.6%, *n* = 77).

56% of respondents reported that their company was headquartered in California (*n* = 225), followed by 5.7% in Minnesota (*n* = 23) and 5.5% in Massachusetts (*n* = 22). All other states had less than 5% representation.

### Company Leadership

**Figure 2:**
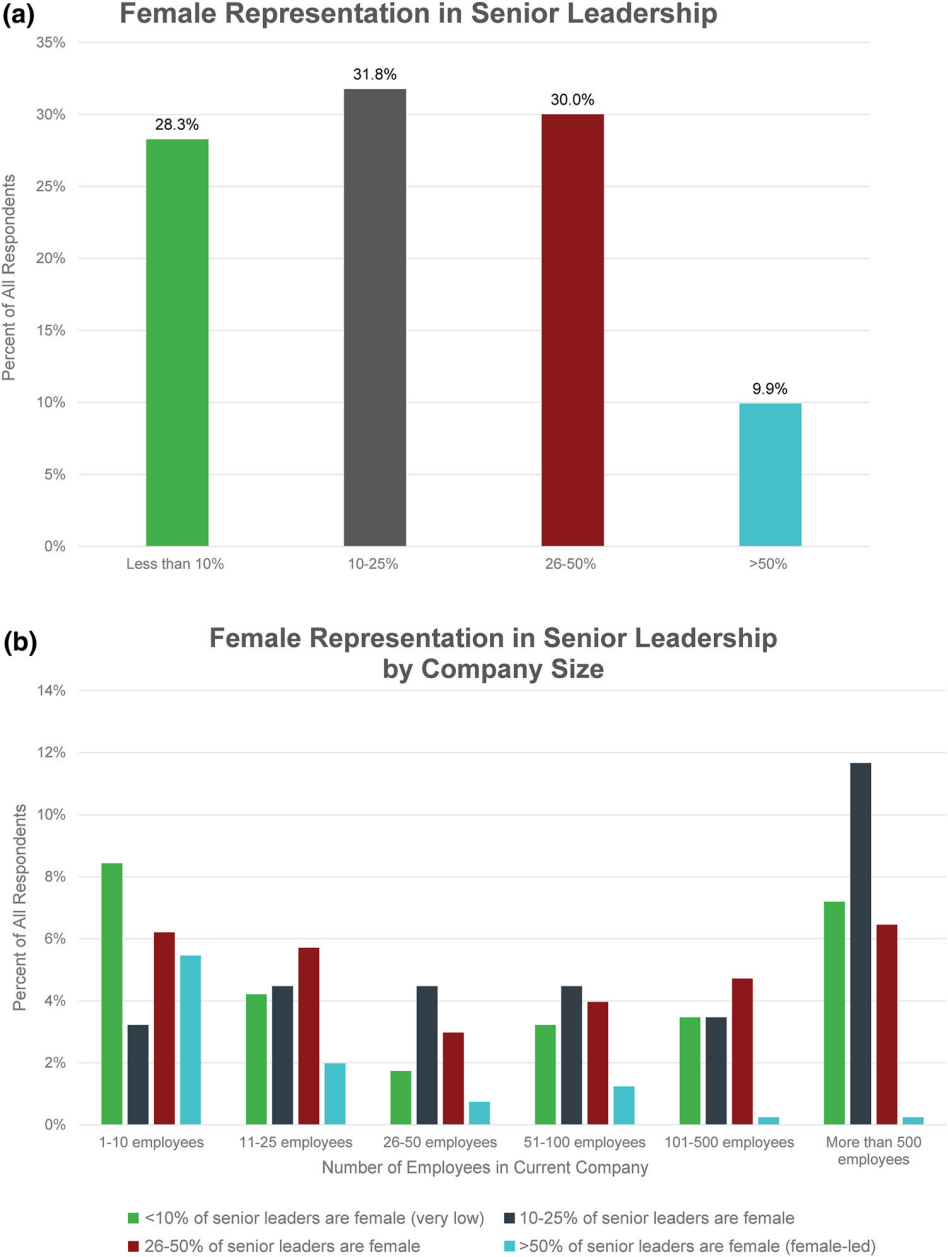
(a) Respondents were asked, “What percentage of senior leaders (director and above) at your current company are female?” and given the listed options from which to choose. (b) This chart shows data from the previous question, organized by company size.

When asked about the gender of senior leaders (directors and above) at their current companies, only 9.9% of respondents (*n* = 40) worked in a company where the majority of leaders were women (Fig. 2a). Of that 9.9%, the majority (55.0%, *n* = 22) worked in small companies (10 employees or less) (Fig. 2b).

### Mentorship

61.0% of female respondents (*n* = 153) and 69.7% of male respondents (*n* = 106) reported that they have or have had a mentor in their health technology career. When asked the gender of their most influential mentor, 86.8% of men (*n* = 92) and 55.6% of women (*n* = 85) cited a male mentor. 69% of all respondents with mentors (*n* = 179) indicated that those connections had been “very helpful” in their careers.

### Job Satisfaction/Inclusive Environment

Male and female respondents were presented with a series of statements that served as proxies for job satisfaction (e.g., “I speak freely at meetings; my professional contributions are heard and valued”) and an inclusive environment (e.g., “The workplace empowers women to reach their full potential”) and asked to indicate whether they agree, disagree, or are neutral/have no opinion.

Across genders, respondent agreement with the proxy statements for job satisfaction and inclusive environment was linked to job retention. Of the 214 respondents who agreed with at least five of the seven proxy statements, only 15.4% were thinking about leaving their jobs. (Note: Five out of seven was subjectively chosen as a stronger indicator than agreement with a simple majority.) Of the 189 respondents who did not agree with five or more of the statements, 40.7% were thinking about making an employment change (*p* < 0.001).

Women responding to the survey were more likely than men to be thinking about leaving their current job. 16.4% (*n* = 25) of men were considering a change compared to 33.9% (*n* = 85) of women (*p* < 0.001).

When those considering a job change were asked why, 44.7% of women (*n* = 38) reported that they were “dissatisfied with management” compared to 8.0% of men (*n* = 2). 42.4% of women (*n* = 36) were “seeking more opportunity for advancement” compared to 8.0% of men (n = 2). Approximately the same percentage of men (32.0%, *n* = 8) and women (32.9%, *n* = 28) were thinking of leaving because they were “seeking more interesting/challenging work.” 40.0% of men (*n* = 10) and 5.9% of women (*n* = 5) cited “other” reasons.

When those considering a job change were asked what they planned to do next, 40.0% of men, (*n* = 10) and 75.3% of women (*n* = 64) reported that they would seek another full-time job in health technology. 32.0% of men (*n* = 8) planned to start their own companies, while only 1.2% of females (*n* = 1) reported this intent. 12% of men (*n* = 3) and 12.9% of women (*n* = 11) planned to work part time or become a consultant.

**Figure 3:**
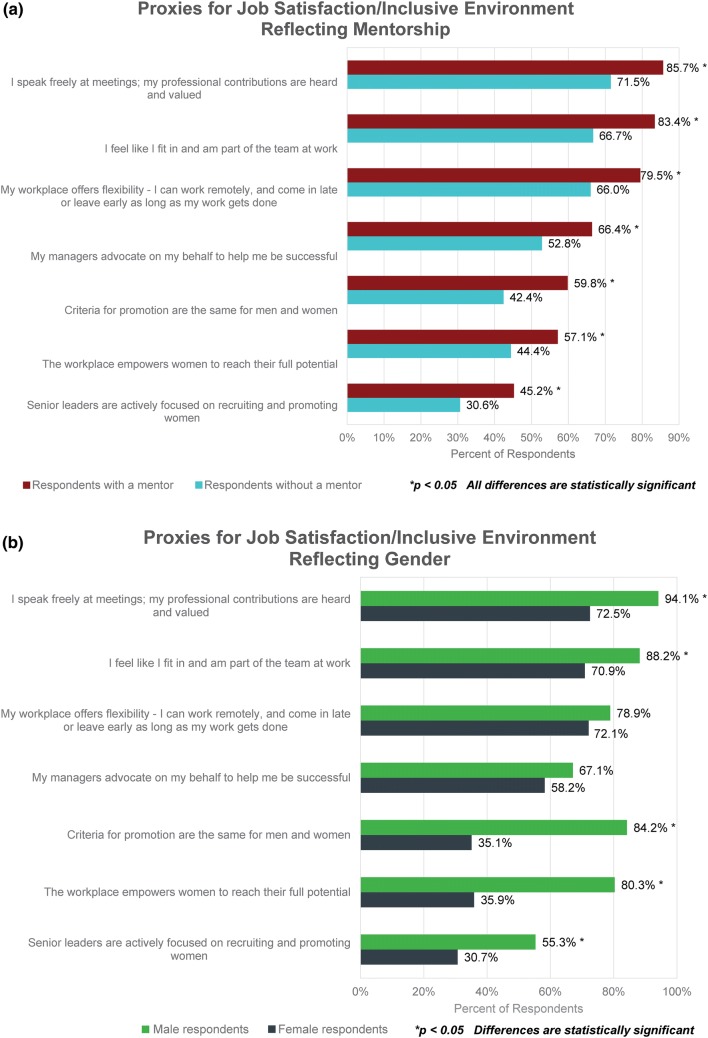
(a) Respondents were asked, “Based on your own experience (across your health tech career), please rate your agreement or disagreement with the following statements.” Possible responses were “agree,” “neutral/no opinion,” and “disagree.” This charts shows the resulting data, organized by whether or not the respondents had a mentor. (b) This chart shows the data from the previous question, organized by respondent gender.

Being male and/or having a mentor were both associated with higher scores on the proxies for job satisfaction and inclusive environment (Figs. 3a and 3b). Male respondents and those with a mentor scored higher on all proxy statements compared to female respondents or those without a mentor. Most differences were significant as indicated by the asterisks (*p* < 0.05).

When we compared women with and without a mentor, women with mentors had significantly higher scores on responses to all job satisfaction/inclusive environment proxies (*p* < 0.05). When we compared men with and without a mentor, men with mentors had significantly higher scores on their response to all job satisfactions proxies (*p* < 0.05). However, having a mentor did not result in any significant increase in men’s scores on proxies related to inclusive environment (e.g., the last three factors listed in Figs. 3a and 3b).

### Perceptions of Equality

**Figure 4:**
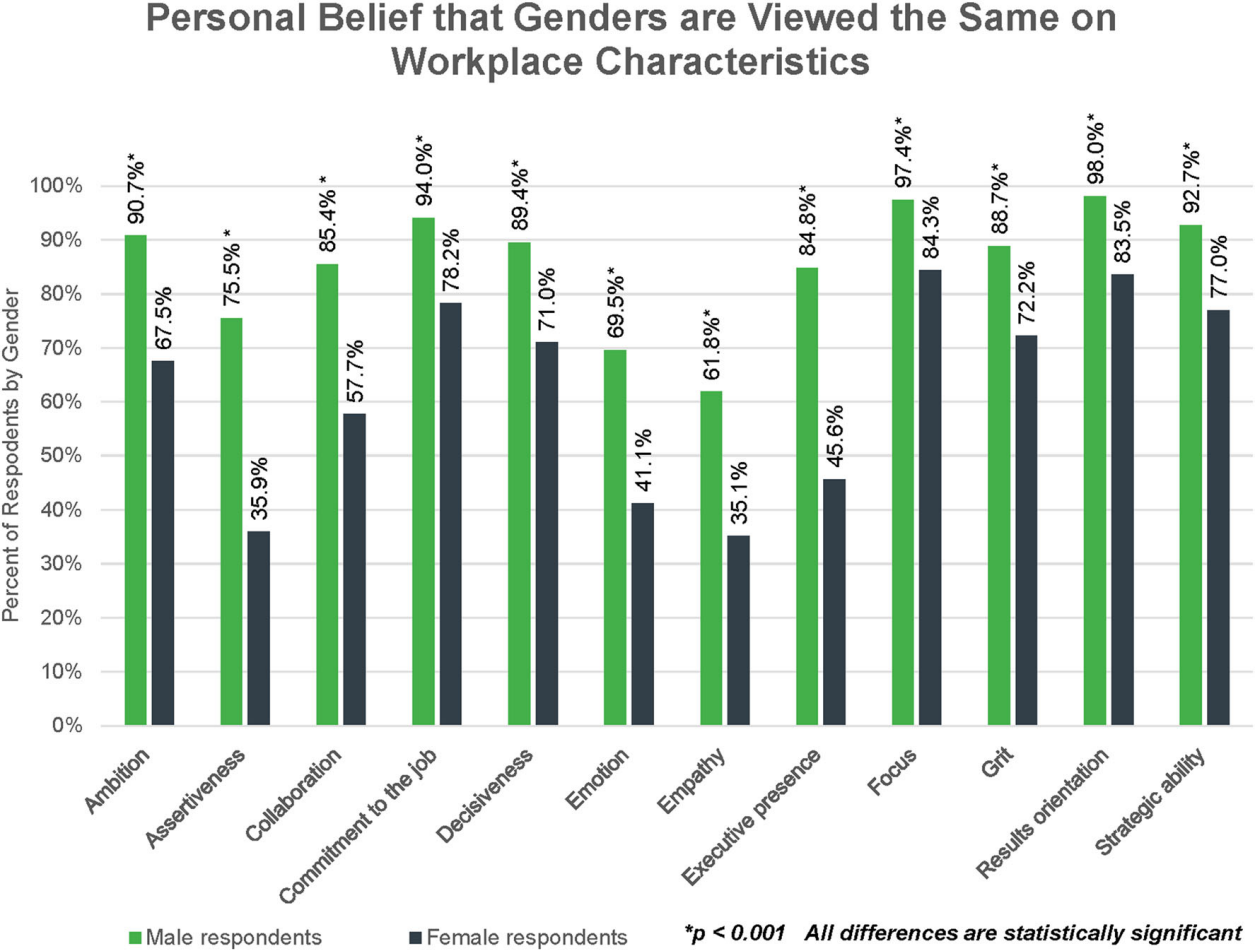
Respondents were asked, “Do you view women and men the same or different with regard to the following characteristics in the workplace?” Possible responses were “viewed about the same” and “viewed differently.” This chart shows data for those who selected “viewed about the same.”

When asked whether they personally viewed men and women as the same or different on a series of workplace characteristics, men were more likely than women to report that they viewed both genders the same, while women reported more variation between the genders. For example, 92.7% of men (*n* = 140) said they viewed men and women as equal in strategic ability whereas only 77.0% of women (*n* = 191) shared the same opinion (Fig. 4). Male and female respondents diverged most in their personal views on the characteristics of assertiveness, emotion, empathy, and executive presence. All responses were statistically significant between male and female responders (*p* < 0.001).

Respondents who indicated that they believed men and women were “viewed differently” for any given characteristic were asked in a secondary question whether they thought that trait applied more to men or women. In general, male and female respondents presented with these follow-up questions agreed in their assessment of which traits applied more to men vs. women, especially on the characteristics of assertiveness, emotion, empathy, and executive presence.

For example, for the characteristic of emotion, 150 men answered the preliminary question. 45 said the genders were “viewed differently” and were presented with the secondary question. 42 assigned emotion to women (93.3%) and 3 assigned that characteristic to men (6.7%). Similarly, 248 women answered the preliminary question and 144 said the genders were viewed differently with regard to emotion. Of those, 141 (97.9%) assigned emotion to women and 3 (2.1%) assigned that characteristic to men. Male and female responses were similar for empathy. The same pattern was present for assertiveness and executive presence, but with both male and female respondents overwhelmingly assigning those characteristics to men.

**Figure 5:**
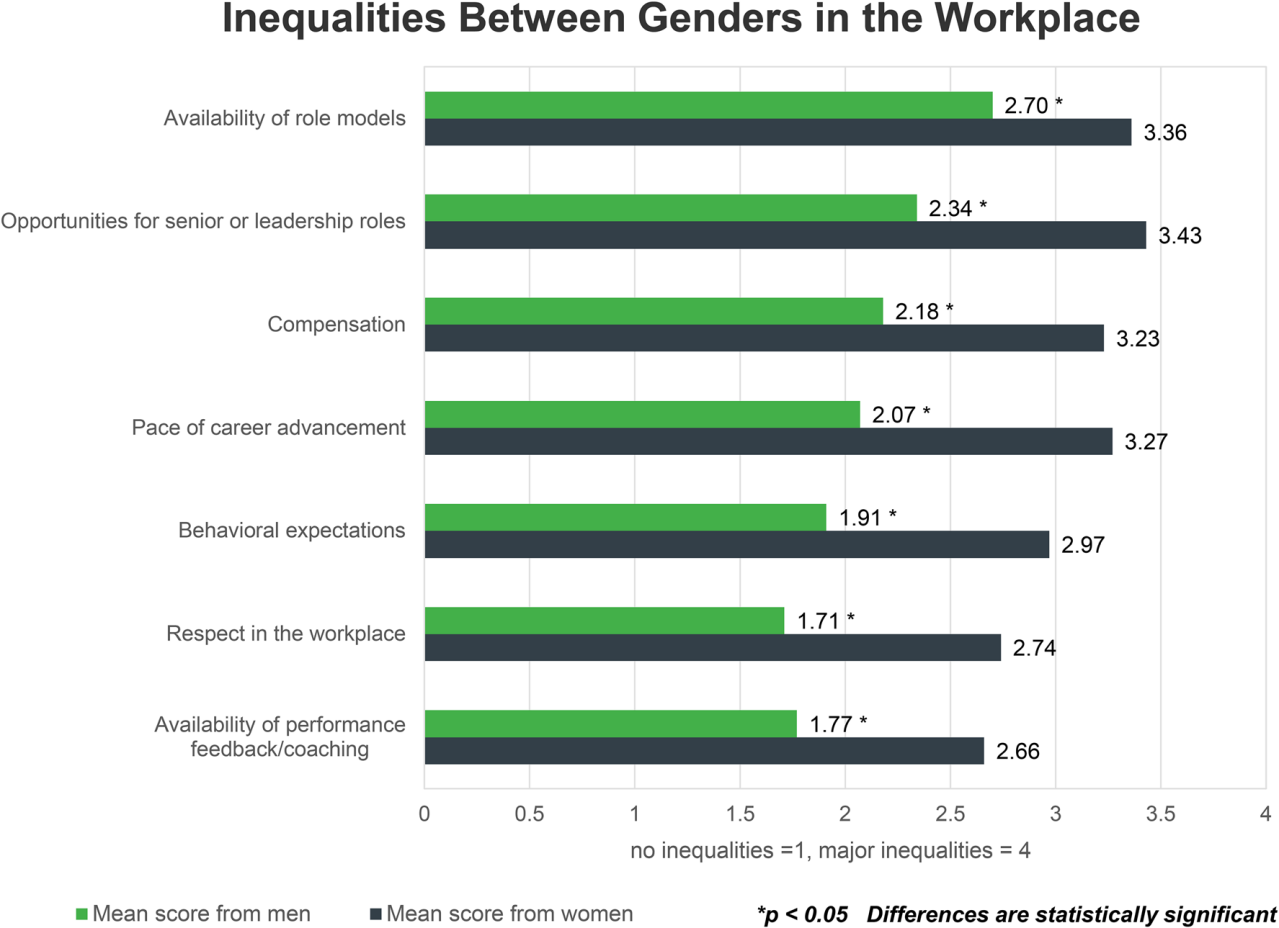
Respondents were asked, “Where do you believe there are the biggest inequalities between genders in health technology?” Choices for each factor were: no inequalities = 1, minor inequalities = 2, moderate inequalities = 3, major inequalities = 4.

Next, respondents were presented with a series of issues and asked to use a four point system to rate where they believed the greatest inequalities exist between men and women in their workplace. Across all issues, men perceived lesser inequalities compared to women at significant levels (Fig. 5; *p* < 0.05).

**Figure 6:**
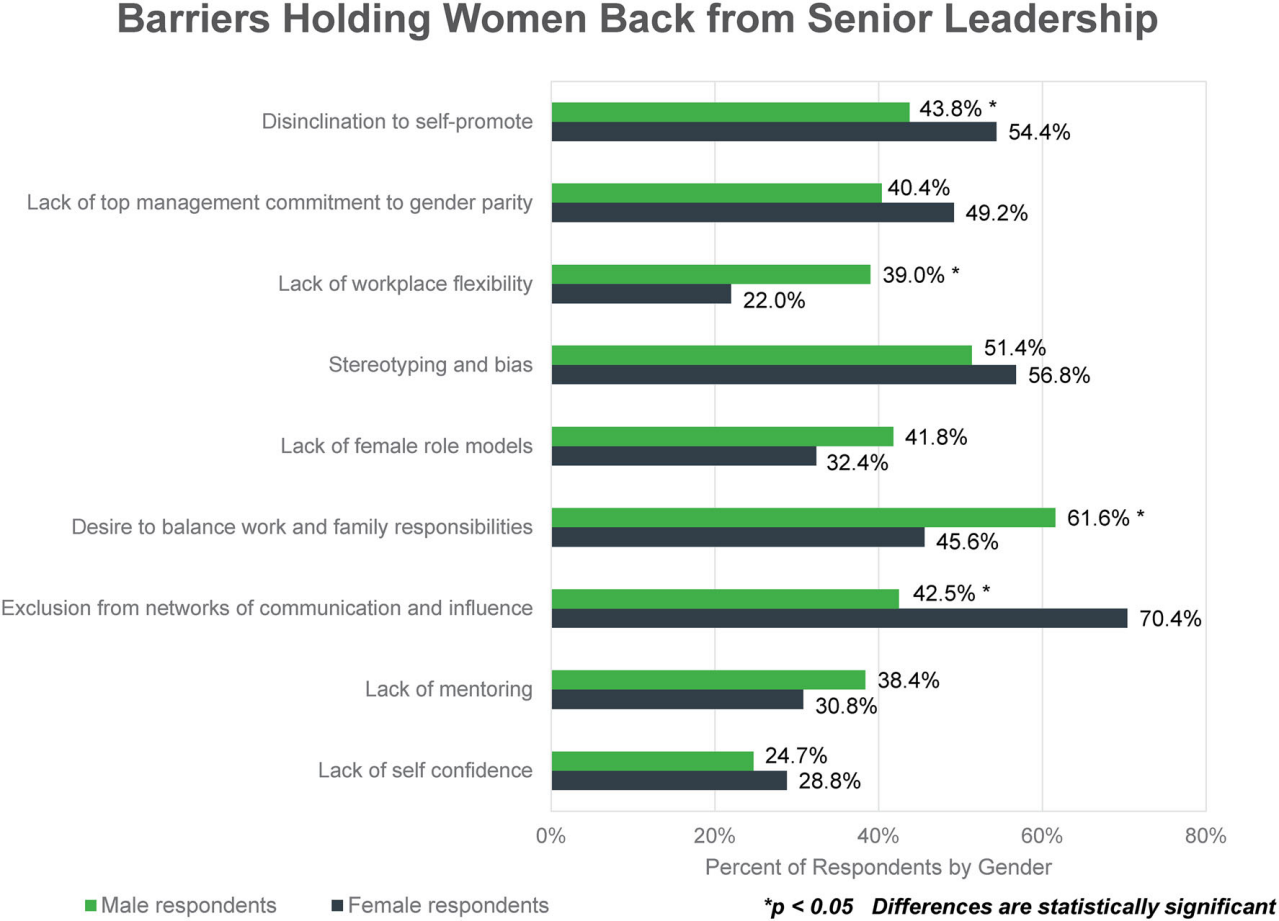
Respondents were asked, “In your view, which barriers hold women back from senior leadership?” and instructed to rank the top four in terms of impact. In the chart, the percent reflects the proportion of respondents who included each barrier in their top four.

A set of barriers with the potential to hold women back from senior leadership roles was included in the survey and respondents were asked to select the top four they believed to be most problematic (Fig. 6). 70.4% of females (*n* = 176) listed “exclusion from networks of communication and influence” as a top concern limiting women from advancing into senior leadership roles compared to 42.5% of men (*n* = 62), while 61.6% of men (*n* = 90) cited “desire to balance work and family” compared to 45.6% of women (*n* = 114).

Respondents were asked if they had observed or experienced gender discrimination while working in health technology. Overall, 53.3% (*n* = 215) responded affirmatively. 72.1% of women (*n* = 181) said that they had observed or experienced gender discrimination, while only 22.4% of men (*n* = 34) answered the same way (*p* < 0.001).

A write-in box provided for respondents to optionally describe the gender discrimination they had observed or experienced garnered 196 responses, with the plurality of comments (49%, *n* = 96) focused around two main themes: (1) bias in hiring or promotion process, and (2) perceptions of qualifications and competence. The next most common theme (22%, *n* = 43) involved not being heard or taken seriously, not having one’s accomplishments acknowledged, or not feeling as though one’s opinion was valued.

Of all respondents, 77.9% (*n* = 314) were in positions that required them to recruit and/or hire other employees. 69.1% of these individuals (*n* = 217) reported that they considered gender balance in their hiring decisions in addition to seeking the most qualified candidate, and 61.4%, (*n* = 134) stated that it was somewhat or very difficult to achieve.

When asked why it was difficult to achieve better gender balance in their company, 86.0% (*n* = 43) of responding males said this was due to a lack of qualified candidates compared to 59.5% of responding females (*n* = 50; *p* = 0.001). 40.4% of responding women (*n* = 34) chose the response “another reason.”

### Work-Life Balance

On the topic of work-life balance, there were significant differences between male and female responses. Only 15.8% of men (*n* = 24) felt that “family responsibilities make it harder to get ahead at work” compared to 29.1% of women (*n* = 73; *p* = 0.002). 32.9% of men (*n* = 50) agreed with the statement “I can start a family and continue to advance at the same pace in my current company as my peers without children” compared to only 17.5% of women (*p* < 0.001).

Only 12.2% of male respondents (*n* = 19) and 19.0% of female respondents (*n* = 47) had taken time off of work—besides maternity/paternity leave—to focus on family. The majority of men reported that it took them less than 6 months to return to work when they were ready (78.9%, *n* = 15), while the majority of women indicated that it took them more than 6 months (58.6%, *n* = 24).

### Fundraising

The last section of the survey focused on fundraising in health technology. A total of 247 respondents (45.7% male, *n* = 113 and 54.3% female, *n* = 134) stated that they had been part of a management team seeking funding. When asked their title when fundraising, there were notable differences by gender. Within this subset of fundraisers, males represented 54.5% of founders/co-founders (*n* = 30), 65.7% of CEOs (*n* = 44), and 29.3% of senior staff (*n* = 27). Women made up 45.5% of founders/co-founders (*n* = 25) in this group, but disproportionately fewer CEOs (34.3%, *n* = 23) and disproportionately more senior staff (70.7%, *n* = 65).

Only 18.0% of women (*n* = 23) and 8.6% of men (*n* = 10) who had been involved in fundraising had pitched to any entities focused on investing in women-led companies. 47.7% of men (*n* = 54) and 81.1% of women (*n* = 103) reported that less than 10% of the investors they met with were female.

When asked if they believed that male and female members of their pitch team were treated differently, only 9.7% of men (*n* = 11) said yes compared to 47.0% of women (*n* = 63; *p* < 0.001).

## Discussion

As noted, gender diversity is linked to catalyzing and sustaining innovation^20^ and increasing the collective intelligence of an organization.^28^ In health technology specifically, gender diversity also enhances the ability to innovate for different patient populations and allows companies to capitalize on the perspectives of women, who are the primary consumers and decision makers for family healthcare.^27^

Given these considerations, along with the relatively widespread presence of women in healthcare jobs, as well as in biomedical engineering and medical pipelines, it might be reasonable to expect that the results of our survey would reflect a more equitable gender landscape in health technology. However, our data highlighted multiple deep-seated problems that affect the experiences of women and have the potential to drive them out of the field.

There are multiple limitations to our study. First, the overall number of survey responses represents a small proportion of the industry, in part reflecting the willingness of participants to complete a 104 question survey. Also our sample is not geographically balanced. California is over represented, with more than 50% of respondents working for companies headquartered there. However, given our interest in understanding gender dynamics in small and mid-sized companies, individuals from California provide an important perspective since the state is known for start-up activity, is a leader in medical device patent filings, and attracts disproportionately more venture capital than anywhere in the US.^5^ Additionally, California’s health technology ecosystem shares traits in common with other US health technology hubs, such as Massachusetts and Minnesota, for example, which also attract substantial life science venture capital funding^5^ and have a high number of medical device employees and medical device patent filings.^23^

Second, the selection of groups to distribute the survey may have biased the responses. Working through trade organizations and specialty mailing lists may have connected us with individuals who are more active in the industry. It also seems that the survey reached a disproportionate number of people working in management roles vs. lower-level positions. However, as noted, identifying and accessing individuals who work specifically in health technology (as opposed to other sectors) can be difficult. At a minimum, these groups provided a reliable channel to members of that target audience.

Third, we are not able to confirm how many responses came to us through each channel or calculate an accurate response rate. Respondents were asked whether they were members of either trade group. 134 reported that they were members of MDMA, and 135 said they were members of AdvaMed. But we did not inquire about membership in MedTechWomen or the Stanford Biodesign Alumni Association. And, as noted, there was likely significant overlap among the mailing lists.

Fourth, question construction could have prejudiced responses, although we attempted to minimize leading questions by trialing them before widespread dissemination. In addition, respondent self-selection may have biased results. In this study, in fact, substantially more women than men participated and it can be argued that those who responded represent a biased perspective. Additionally, the survey did not collect data on race or ethnicity. However, it is widely understood that those who are underrepresented by race and/or ethnicity can have even more challenging experiences in the workplace.^18^

Finally, self-reported data are inherently subjective. However, the primary goal of this study was to characterize the subjective experience of health technology professionals on gender-related issues. Recording perceptual data is a well-established method of assessing unconscious gender bias at work.^4^

Despite these limitations, the survey results shed light on two especially critical issues: the underrepresentation of women in senior leadership and striking differences in male and female perceptions of and experiences in the health technology workplace.

### Women in Senior Leadership

The survey results suggest that there is a major gender imbalance in the senior leadership ranks of health technology companies. A majority of respondents work in organizations where women make up one-quarter or less of the senior leadership team. Only 10% are employed in companies with more than half female senior leaders (Fig. 2a). Furthermore, these data potentially exaggerate the presence of female senior leaders in health technology because a majority of these respondents are from companies with 10 employees or less (Fig. 2b).

Respondents pointed to multiple barriers that prevent women from reaching senior leadership (Fig. 6). Respondents of both genders cite stereotyping/bias as a major barrier. However, while women acknowledge that the desire to balance workplace and family responsibilities can hinder advancement, they point to exclusion from networks of communication/influence as the most significant problem. Their male counterparts underrate exclusion from networks and perceive the desire for work-life balance as the greatest obstacle for women on the path to senior leadership. This can become a self-fulfilling problem when male leaders governing work assignments assume that female employees are not interested in or able to take on new tasks or leadership roles due to their family responsibilities and, therefore, never offer them the chance.^13^

Female respondents also believe that disinclination to self-promote, a strategy generally considered necessary for professional advancement,^2^ is a barrier to women moving into senior leadership roles at a significantly higher level than male respondents. There are many reasons why women may be hesitant to advocate for themselves.^2^,^12^ One explanation suggested by our data is that they may be less secure in the workplace than their male counterparts (Fig. 3b). More women than men reported feeling unsure speaking freely at meetings and that their contributions are valued. Even more importantly, women are less likely than men to perceive that promotion criteria are equitable across genders, their workplace is empowering them to reach their full potential, and that the senior leaders at their companies are focused on recruiting and promoting women. Taken in combination, these factors could be discouraging to women with senior leadership aspirations and may drive them to change jobs. More than one-third of female respondents are thinking about leaving their current positions, and the primary reasons involve dissatisfaction with management and a desire for greater advancement opportunities.

Other areas flagged by both genders where major inequalities exist were the availability of role models, the pace of career advancement, and compensation (Fig. 5). Without role models to demonstrate that women can, in fact, rise to the top, upward mobility can feel slow and/or unattainable to female employees.^6^,^8^ Similarly, compensation is a well-documented challenge for working women in the US across sectors.^11^ Substantial research indicates that the disparity in entry level compensation initiates a trend of unequal pay for equal work that can persist across a woman’s entire career.^9^,^18^

### Male and Female Experiences in the Workplace

Overall, men responding to the survey believe that the work environment for their female colleagues is more meritocratic and inclusive than the women report. The data indicate that women experience a non-inclusive culture that, for the most part, their male colleagues may not even see. For example, far fewer female than male respondents believe that they fit in and are part of the team at work (Fig. 3b). And while men largely report that the genders are viewed the same on workplace characteristics, women perceive this to be the case at much lower rates (Fig. 4). One area where men and women *do* seem to agree is that gender stereotypes in the workplace are alive and well, with traits such as emotion, empathy, and collaboration assigned to women and assertiveness, executive presence, and ambition more often reserved for men.

Male respondents also are more likely to believe that diversity and inclusion efforts are sufficient and/or the problem is not that great, whereas women are more likely to believe more improvements are needed (Fig. 3b). For instance, more than half of male respondents report that senior leaders are actively focused on recruiting and promoting women within their companies compared to less than one-third of females. Well over three-quarters of men believe that promotion criteria are equitable compared to just over one-third of women. Only 10% of responding men believe that male and female members of fundraising teams are treated differently compared to nearly half of responding women. Similar findings are evident across industries and consistent with other studies.^18^

### Moving Forward

Our data come from a detailed survey with more than 400 responses from non-financially-incentivized participants who work specifically for health technology organizations. As such, they provide a credible first-look at the current state of gender diversity in the health technology field.

The results are provocative and spotlight important issues, including the extent to which women are underrepresented in senior leadership and how vastly different men and women experience the workplace. They also provide more nuanced insights that should be used to fuel larger, more rigorous studies of gender equality in health technology. Minimally, the data should signal to health technology companies and training programs that our field is not immune to the gender-related issues that negatively affect the high technology industry, and that proactive steps to assess and improve their work environments on diversity and inclusivity may prevent them from losing talented female contributors.

Major players in health technology have begun addressing issues of gender (and racial) diversity. As one example, Medtronic, the largest medical device company, has a Global Inclusion, Diversity, and Engagement team tasked with ensuring that, over time, the company’s workforce reflects the racial and gender makeup of the communities in which it is based.^16^ Other large companies, such as Johnson & Johnson, Edwards Lifesciences, and Abbott, have launched similar initiatives. While more time is needed to determine their effectiveness in changing corporate culture, they are significant in that they reflect a groundswell in acknowledging the importance of diversity in achieving desired business results. That said, this issue must not be left to a few large players to address. Organizations of all sizes must be engaged to make a meaningful difference in improving the gender landscape in health technology.

Importantly, academic programs focused on health technology innovation are in a unique position to help drive change from the bottom up. By increasing awareness and understanding of gender stereotypes and unconscious bias among our trainees, universities can equip future health technology leaders to build more inclusive workplaces as they go forward in their careers. Sharing data from this and other relevant studies is one way to engage students on the topic. Another is to leverage our project-based courses/programs to model desired behavior, help students create inclusive teams, and challenge them to practice behaviors that enable all team members to achieve their full potential. Additionally, academia can lead more studies to make sure our understanding of key issues is sound and that resulting improvement initiatives truly will make a difference.

The benefits associated with increased diversity and inclusion are too great to be ignored. However, the results of our study indicate that current efforts around gender diversity and inclusion in health technology are not having a sufficient effect. Through greater awareness, evaluation, and action, health technology has the opportunity to lead other sectors in achieving these desired results.

## References

[1] Association of American Medical Colleges. The majority of U.S. medical students are women, new data show, December 10, 2019. https://www.aamc.org/news-insights/press-releases/majority-us-medical-students-are-women-new-data-show.

[2] Ballakrishnen S, Fielding-Singh P, Magliozzi D (2018). Intentional invisibility: professional women and the navigation of workplace constraints. Sociol. Perspect..

[3] Berlin, G., L. Darino, M. Greenfield, and I. Starilova. Women in the healthcare industry. McKinsey & Company, June 2019. https://www.mckinsey.com/industries/healthcare-systems-and-services/our-insights/women-in-the-healthcare-industry.

[4] Breaking barriers: unconscious gender bias in the workplace. International Labour Organization, August 2017. https://www.ilo.org/actemp/publications/WCMS_601276/lang–en/index.htm.

[5] California Life Sciences Industry Report 2019. California Life Sciences Association and PWC. https://califesciences.org/wp-content/uploads/2018/11/clsa-2019-california-life-sciences-industry-report.pdf.

[6] D’Armiento J, Witte S, Dutt K (2019). Achieving women’s equality in academic medicine: challenging the standards. The Lancet..

[7] Diversity in high tech. U.S. Equal Employment Opportunity Commission, May 2016. https://www.eeoc.gov/eeoc/statistics/reports/hightech/.

[8] Fernando D, Cohen L, Duberley J (2018). What helps? Women engineers’ accounts of staying on. Hum. Resour. Manag. J..

[9] Freund K, Raj A, Kaplan S (2016). Inequities in academic compensation by gender: a follow-up to the National Faculty Survey Cohort study. Acad. Med..

[10] Funk, C., and K. Parker. Women and men in STEM often at odds over workplace equity. Pew Research Center, January 9, 2018. https://www.pewsocialtrends.org/2018/01/09/women-and-men-in-stem-often-at-odds-over-workplace-equity.

[11] Graf, N., A. Brown, and E. Patten. The narrowing, but persistent, gender gap in pay. Fact-Tank News in the Numbers, Pew Research Center. March 22, 2019. https://www.pewresearch.org/fact-tank/2019/03/22/gender-pay-gap-facts/.

[12] Greenberg C (2017). Association for Academic Surgery presidential address: sticky floors and glass ceilings. J. Surg. Res..

[13] Halley M, Rustagi A, Torres J (2018). Physician mothers’ experience of workplace discrimination: a qualitative analysis. BMJ.

[14] Haskins, J. Where are all the women in surgery? AAMC New, July 15, 2019. https://www.aamc.org/news-insights/where-are-all-women-surgery.

[15] Hill, C., C. Corbett, and A. St. Rose. Why so few? American Association of University Women, 2010. https://www.aauw.org/aauw_check/pdf_download/show_pdf.php?file=why-so-few-research.

[16] Inclusion and diversity highlights from fiscal year 2018. Medtronic. https://www.medtronic.com/us-en/about/citizenship/supporting-a-global-workforce/inclusion-diversity.htm.

[17] Johns M (2013). Breaking the glass ceiling: structural, cultural and organizational barriers preventing women from achieving senior and executive positions. Perspect Health Inf Manag..

[18] Krikovich, A., K. Robinson, I. Starikova, *et al*. Women in the workplace 2017. McKinsey & Company, October 2017. https://www.mckinsey.com/featured-insights/gender-equality/women-in-the-workplace-2017.

[19] Labor force statistics from the current population survey. United States Department of Labor, January 18, 2019. https://www.bls.gov/cps/cpsaat11.htm.

[20] Lorenzo, R., N. Voigt, *et al*. How diverse leadership teams boost innovation. Boston Consulting Group, January 23, 2018. https://www.bcg.com/en-us/publications/2018/how-diverse-leadership-teams-boost-innovation.aspx.

[21] McDonagh KJ, Bobrowski P, Hoss M (2014). The leadership gap: ensuring effective leadership requires inclusion of women at the top. OJL.

[22] Professionally active physicians by gender. Kaiser Family Foundation State Health Facts, March 2019. https://www.kff.org/other/state-indicator/physicians-by-gender/.

[23] Shepard, M. A Snapshot of the Hottest U.S. Medical Device Regions. Medical Product Outsourcing, May 5, 2019. https://www.mpo-mag.com/issues/2019-05-01/view_columns/a-snapshot-of-the-hottest-us-medical-device-regions/.

[24] Silicon Valley’s sexism problem. The Economist, April 15, 2017. https://www.economist.com/leaders/2017/04/15/silicon-valleys-sexism-problem.

[25] Williams J, Phillips K, Hall E (2016). Tools for change: boosting the retention of women in the STEM pipeline. J. Res. Gender Stud..

[26] Women in the labor force: a databook. U.S. Bureau of Labor Statistics. December 2018 | Report 1077. https://www.bls.gov/opub/reports/womens-databook/2018/pdf/home.pdf.

[27] Women, work and family health: key findings from the 2017 Kaiser Women’s Health Survey. Henry J. Kaiser Family Foundation, March 13, 2018. https://www.kff.org/womens-health-policy/issue-brief/women-work-and-family-health-key-findings-from-the-2017-kaiser-womens-health-survey/.

[28] Woolley A, Chabris C, Pentland A (2010). Evidence for a collective intelligence factor in the performance of human groups. Science..

[29] Yoder, B.L. Engineering by the numbers. ASEE, 2017. https://www.asee.org/documents/papers-and-publications/publications/college-profiles/2017-Engineering-by-Numbers-Engineering-Statistics.pdf.

[30] Young C, Abrnousi F, Rzeszut A (2019). Sex differences in the pursuit of interventional cardiology as a subspecialty among cardiovascular fellows-in-training. J. Am. Coll. Cardiol. Interv..

